# M2 macrophage exosomes reverse heart failure post-myocardial infarction by suppressing type 1 interferon signaling in myeloid cells

**DOI:** 10.1016/j.ymthe.2025.10.010

**Published:** 2025-10-04

**Authors:** Martin Ng, Alex S. Gao, Tuan Anh Phu, Ngan K. Vu, Robert L. Raffai

**Affiliations:** 1 Department of Veterans Affairs, Surgical Service (112G), San Francisco Veterans Affairs Medical Center, San Francisco, CA 94121, USA; 2 Northern California Institute for Research and Education, San Francisco, CA 94121, USA; 3 Department of Surgery, Division of Vascular and Endovascular Surgery, University of California, San Francisco, San Francisco, CA 94143, USA

**Keywords:** M2 human macrophage exosome, myocardial infarction, heart failure, type 1 interferon, immunosuppression, CD11b^+^ myeloid cell, therapeutic exosome, extracellular vesicles

## Abstract

Effective treatment strategies to alleviate heart failure that develops as a consequence of myocardial infarction (MI) remain an unmet need in cardiovascular medicine. In this study, we uncover that exosomes produced by human Tohoku Hospital Pediatrics-1 (THP-1) macrophages cultured with the cytokine interleukin-4 (THP1-IL4-exo) reverse cardiac functional decline in mice that developed MI in response to diet-induced occlusive coronary atherosclerosis. The therapeutic benefits of THP1-IL4-exo stem from their ability to drive transcriptional reprogramming of inflammatory responses in myeloid cells. Notably, repeated infusions of THP1-IL4-exo led to the suppression of type 1 interferon signaling in circulating Ly-6C^hi^ monocytes as well as in myeloid cells within the bone marrow and cardiac tissue. *In vitro* studies with primary macrophages stimulated with double-stranded DNA confirmed an ability for THP1-IL4-exo to confer suppression of type 1 interferon-mediated immune activation and inflammation. Collectively, these benefits contribute to the control of myelopoiesis, recruitment of cardiac myeloid cells, and preservation of populations of resident cardiac macrophages that together mitigate cardiac inflammation, adverse ventricular remodeling, and heart failure. Our findings introduce THP1-IL4-exo, one form of M2-macrophage exosomes, as novel anti-inflammatory and tissue repair therapeutics to preserve cardiac function post-MI.

## Graphical abstract

**Figure:**
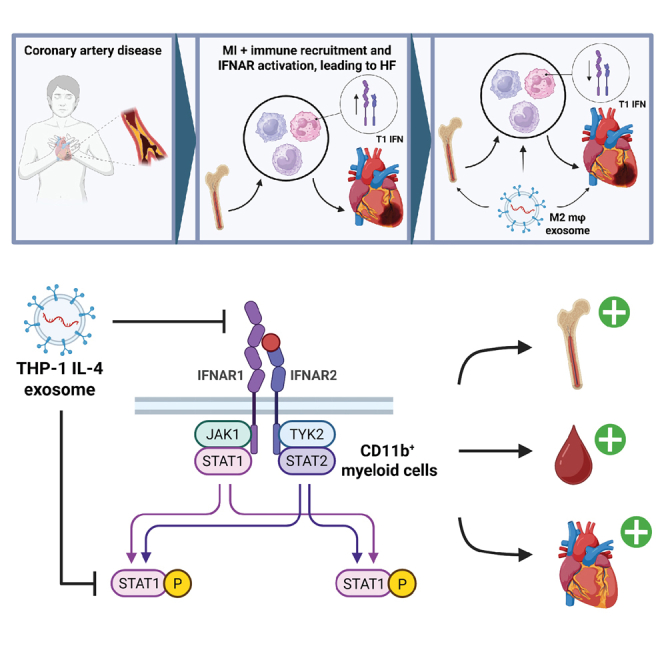

## Teaser

This study reports that exosomes produced by interleukin-4-polarized THP-1 human macrophages (THP1-IL4-exo) can serve to reverse cardiac functional decline in mice with heart failure caused by diet-induced myocardial infarction (MI). Ng and colleagues identify type 1 interferon signaling as a key pathway attenuated by THP1-IL4-exo, responsible for conferring cardioprotection post-MI.

## Introduction

Heart failure (HF) remains a leading cause of morbidity and premature death worldwide, with a significant portion of these events resulting from hyperlipidemia-driven cardiovascular disease (CVD).^1^ Occlusive coronary circulation, including via thrombosis due to atherosclerotic plaque rupture, results in cardiac ischemia and myocardial infarction (MI).^2^ While survival rates from MI have improved owing to advances in interventional cardiology, long-term outcomes remain poor due to the onset of HF.^3^ Ischemic myocardial injury is increasingly recognized to orchestrate adverse cardiac tissue remodeling, resulting in ventricular stiffening, dilation, and ultimately, HF with reduced ejection fraction (HFrEF).^4^ Sustained immune reactions represent central elements to adverse cardiac tissue remodeling^5^ and thus offer opportunities for targeted interventions to mitigate and even reverse HFrEF in response to MI.^6^

Initial waves of infiltrating bone marrow (BM)-derived leukocytes, including monocytes, into ischemic cardiac tissue in response to MI have long been recognized as a source of tissue repair.^7^ However, exaggerated cardiac immune cell recruitment sustains inflammation that results in adverse ventricular remodeling and cardiac functional decline.^8^ Recent studies have identified the type 1 interferon (IFN) signaling axis as a critical element responsible for adverse cardiac remodeling and HF in response to MI.^9^ Cytosolic DNA sensors in phagocytic macrophages, including cyclic guanosine monophosphate-adenosine monophosphate synthase-stimulator of IFN genes (cGAS-STING), recognize double-stranded DNA (dsDNA) released as a damage-associated molecular pattern (DAMP) during ischemic cardiomyocyte cell death, a hallmark of MI.^10^^,^^11^ In response, phagocytic macrophages activate the IFN regulatory factor 3 (IRF3) transcription factor, which in turn triggers the type 1 IFN signaling cascade, resulting in the upregulated expression of IFN-stimulated genes (ISGs) that sustain cardiac myeloid cell recruitment, inflammation, and maladaptive ventricular remodeling.^9^ Furthermore, the type 1 IFN signaling cascade was found to be triggered within the BM via sympathetic responses to MI, accentuating myelopoiesis and inflammatory myeloid cell recruitment to cardiac tissue.^12^^,^^13^ Inhibiting components of the type 1 IFN axis mitigates undesirable immune reactions during cardiac tissue repair post-MI, supporting the targeting of this inflammatory signaling axis as a therapeutic strategy for HF.^9^^,^^14^

Extracellular vesicles, including exosomes, are increasingly recognized as sources of intercellular communication, owing to their capacity to transport microRNAs, proteins, metabolites, and other bioactive molecular cargo to recipient cells.^15^^,^^16^^,^^17^ Studies, including those from our laboratory, have reported on macrophage-derived exosomes as mediators of immunometabolic signaling in cardiovascular inflammation and diabetes.^17^^,^^18^^,^^19^ While exosomes produced by macrophages polarized into an M1-like pro-inflammatory state drive inflammatory signaling in the cardiovascular system,^18^ those produced by anti-inflammatory M2-like macrophages suppress nuclear factor κB (NF-κB) signaling and foster the resolution of inflammation and drive atherosclerosis lesion stabilization.^19^ Outcomes of our recent studies on exosomes produced by interleukin-4 (IL-4) polarized human Tohoku Hospital Pediatrics-1 (THP-1) macrophages (THP1-IL4-exo) highlighted their capacity to control cardiometabolic inflammation, improve insulin resistance, and normalize blood glucose levels in obese diabetic mice fed a lipid-rich diet.^17^

Building on these observations, we sought to test the effectiveness of THP1-IL4-exo in suppressing maladaptive cardiac remodeling in our previously described mouse model of diet-induced occlusive coronary atherosclerosis, MI, and HFrEF^20^^,^^21^ Our findings uncover the capacity for THP1-IL4-exo to exert profound cardioprotection and recovery of cardiac function in mice with HFrEF caused by MI. Cardioprotective properties of THP1-IL4-exo stem from their control of type 1 IFN signaling and expression of ISGs in myeloid cells residing within the BM, circulation, and cardiac tissue. Collectively, our findings introduce the therapeutic potential of THP1-IL4-exo, a type of M2-macrophage exosome, in reversing cardiac functional decline and HFrEF that develops in response to MI caused by occlusive coronary atherosclerosis.

## Results

### Study design utilizing ApoE^h/h^/SR-B1^−/−^/Mx1-Cre^+^ mice and biophysical parameters of THP1-IL4-exo

In this study, 20- to 24-week-old male ApoE^h/h^/SR-B1^−/−^/Mx1-Cre^+^ mice were fed an atherogenic Paigen diet for 4.5 weeks to induce hyperlipidemia, occlusive coronary atherosclerosis, and MI, as we previously reported (Figure S1A).^20^^,^^21^ At time point B, mice were switched to a chow diet and intraperitoneally (IP) injected twice over 2 days with 250 μg polyinosinic:polycytidylic acid (pIpC) to activate the Mx1-Cre recombinase that repairs the hypomorphic *Apoe*^h/h^ (HypoE) alleles. This restores physiological ApoE expression in the liver, which rapidly normalizes plasma lipid levels, as we previously reported.^22^ Subsequently, mice were left to recover from MI while undergoing tri-weekly IP treatments with 10^10^ exosomes derived from THP-1 cells exposed to IL-4 (THP1-IL4-exo) or saline (Figure S1A). At time points A, B, and C, blood was collected to determine cholesterol levels and the numbers of monocytes and neutrophils in circulation, while high-resolution ultrasound served to record the parameters of cardiac function. Figure S1B shows the efficacy of Cre-mediated gene repair of the HypoE alleles and chow diet restoration in normalizing plasma cholesterol levels by time point C. Figure S1C further highlights the normalized plasma cholesterol levels among both treatment groups of mice by time point C, the time of tissue collection.

THP1-IL4-exo used in this study were produced and isolated via cushioned-density gradient ultracentrifugation (C-DGUC)^23^^,^^24^ and confirmed to have similar concentrations, size distributions, and morphologies (Figures S2A–S2D), as we previously reported.^17^ Similarly, when examined for microRNA cargo, THP-1-IL4-exo were consistently found to be enriched with miR-146b/378a, but also with miR-23a/b, a pair of microRNAs recognized to control cGAS-STING signaling in myeloid cells (Figure S2E).^25^ To assess their biodistribution in this mouse model, THP1-IL4-exo were labeled with 1,1′-dioctadecyl-3,3,3′3′ tetramethylindotricarbocyanine iodide (DiR [DilC18(7)]) and injected IP into ApoE^h/h^/SR-B1^−/−^/Mx1-Cre^+^ mice. The mice were given one injection of 10^10^ DiR-labeled THP1-IL4-exo or PBS. Six hours post-treatment, the presence of DiR^+^ exosomes was detected in the blood, tibias, femurs, and hearts of the mice (Figures S1D and S1E), as we previously reported in the study of obese diabetic mice.^17^

### THP1-IL4-exo treatments improve left ventricle function and reverse HFrEF post-MI

**Figure 1:**
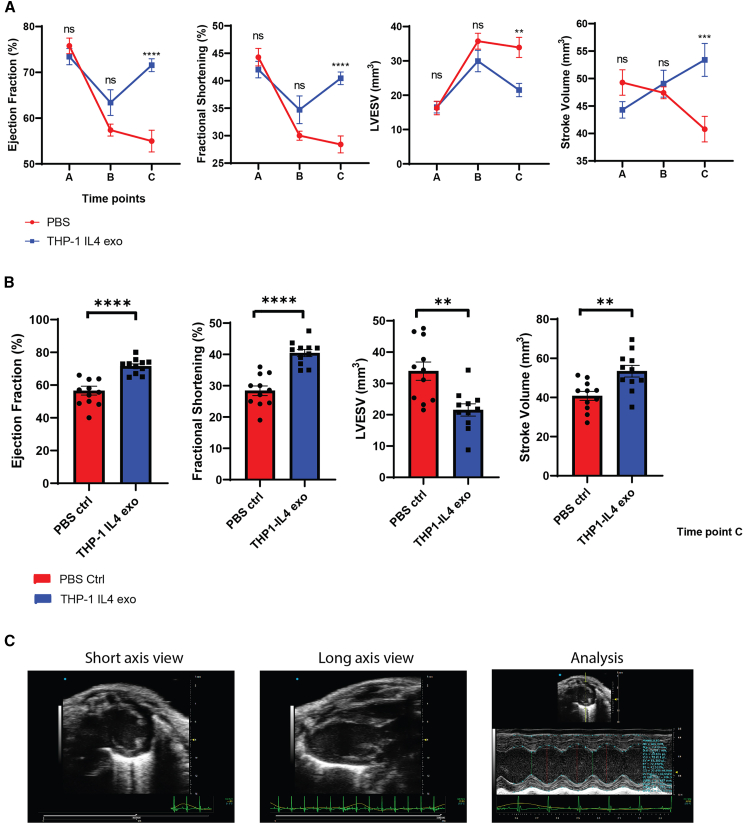
THP1-IL4-exo treatments improve left ventricular function and reverse HFrEF post-MI (A) Echocardiographic measures of left ventricular (LV) performance over the three time points A, B, and C, including ejection fraction (EF), fractional shortening (FS), left-ventricular end systolic volume (LVESV), and stroke volume (SV); data pooled from two independent experiments; *n* = 11 per group. (B) EF, FS, LVESV, and SV measured at time point C; data pooled from two independent experiments; *n* = 11 per group. (C) Representative echocardiographic images of the short axis view (left), the long axis view (center), and the quantitative data analysis of chamber size dimensions (right). ∗*p* < 0.05, ∗∗*p* < 0.01, ∗∗∗*p* < 0.001, and ∗∗∗∗*p* < 0.0001 as determined using two-way ANOVA or unpaired Student’s *t* test. Data are represented as mean ± SEM.

To begin investigating the cardioprotective properties of THP1-IL4-exo, we tested their impact in mitigating cardiac functional decline post-MI. We did so by performing serial echocardiographic measurements of left ventricular (LV) function, including ejection fraction (EF), fractional shortening (FS), LV end-systolic volume (LVESV), and stroke volume (SV) in mice of both treatment groups at all three time points (A, B, and C). As shown in Figure 1A, all mice experienced an expected decline in LV function between time points A and B, corresponding to the period of Paigen diet consumption, which results in occlusive coronary atherosclerosis, MI, and onset of HFrEF, as reported in our prior studies of the model.^20^^,^^21^ Following HypoE allele repair and diet change at time point B, tri-weekly IP injections of THP1-IL4-exo led to significantly improved LV function in mice by time point C, at which numerous parameters of cardiac function were detected to return to baseline levels prior to diet initiation (Figure 1B). Data in Figure 1B show improved LV function parameters, including EF, FS, LVESV, and SV, in mice treated with THP1-IL4-exo at time point C after 4.5 weeks, indicating a reversal of LV functional decline compared to sham-treated mice. Figure 1C shows representative echocardiographic images of the short (left) and long axes (center) of the heart, including a representative image of the quantitative analysis of chamber size dimensions (right). Overall, the cardioprotective benefits of THP1-IL4-exo likely contributed to improve cardiac function post-MI (Figure S1F).

### THP1-IL4-exo treatments suppress the expression of pro-inflammatory and tissue remodeling genes while enhancing the expression of those involved in anti-inflammatory activity in cardiac tissue post-MI

**Figure 2:**
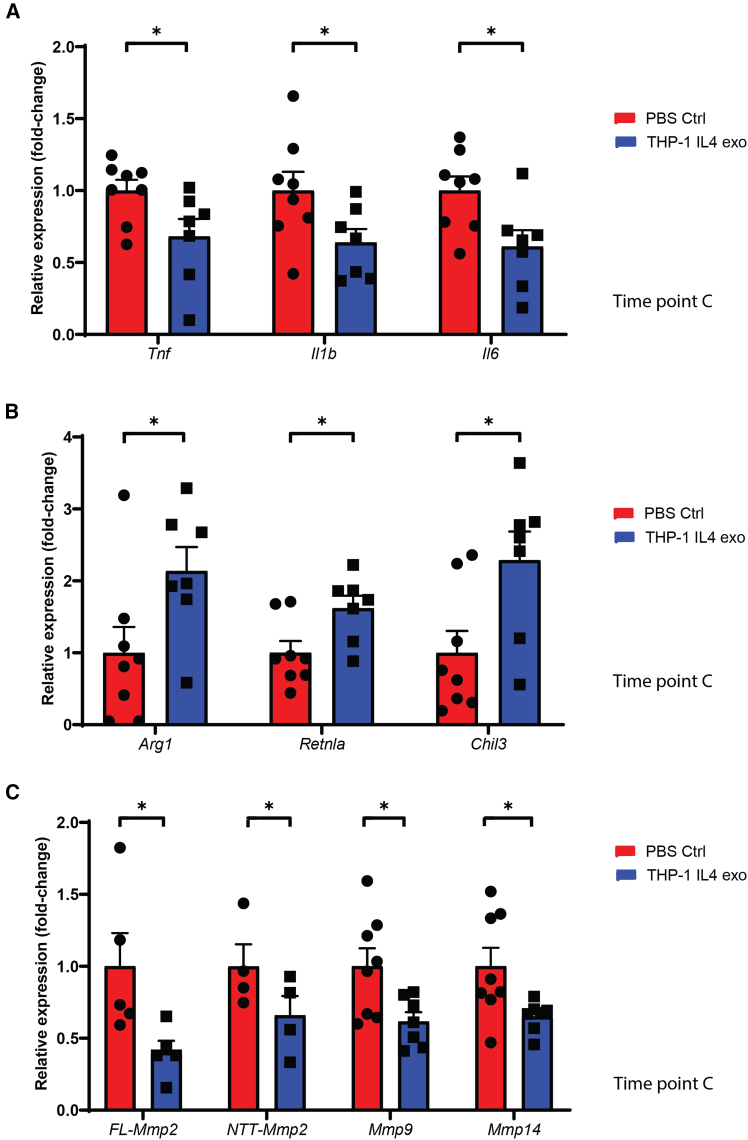
THP1-IL4-exo treatments suppress the expression of pro-inflammatory and matrix metalloproteinase genes while enhancing the expression of M2-like macrophage genes involved in anti-inflammatory activity in cardiac tissue post-MI (A) RT-qPCR analysis of *Tnf*, *Il-1β*, and *Il-6* mRNA expression in whole-heart tissue from mice treated with PBS vs. THP1-IL4-exo, collected at time point C. Gene expression was normalized to *B2m* and *Gapdh* mRNA and presented relative to control; data pooled from two independent experiments; *n* = 7–8 per group. (B) RT-qPCR analysis of *Arg1*, *Retnla*, and *Chil3* mRNA expression in whole-heart tissue from mice treated with PBS vs. THP1-IL4-exo, collected at time point C. Gene expression was normalized to *B2m* and *Gapdh* mRNA and presented relative to control; data pooled from two independent experiments; *n* = 7–8 per group. (C) RT-qPCR analysis of *FL-Mmp2* (full length), *NTT-Mmp2* (N-terminal truncated), *Mmp9*, and *Mmp14* mRNA expression in whole-heart tissue from mice treated with PBS vs. THP1-IL4-exo, collected at time point C. Gene expression was normalized to *B2m* and *Gapdh* mRNA and presented relative to control; data from one experiment (*n* = 4–5 per group) or pooled from two independent experiments (*n* = 7–8 per group). ∗*p* < 0.05 as determined using unpaired Student’s *t* test. Data are represented as mean ± SEM.

To further explore the cardioprotective properties of THP1-IL4-exo, we tested their capacity to suppress inflammation in whole cardiac tissue. Immunosuppression by THP1-IL4-exo was evidenced by the reduced expression levels of inflammatory cytokines, including *Tnf*, *Il1b*, and *Il6* (Figure 2A). In contrast, the expression of genes associated with anti-inflammatory and tissue repair activities, including *Arg1*, *Retnla*, and *Chil3*, were noted to be significantly upregulated (Figure 2B). Furthermore, treatments with THP1-IL4-exo also led to significant reductions in the expression of matrix metalloproteinases (MMPs) that included both full-length (FL) and N-terminal truncated (NTT) *Mmp2*, along with *Mmp9* and *Mmp14*, which are recognized for contributing to adverse cardiac remodeling and ventricular dilation (Figure 2C).^26^^,^^27^^,^^28^

### THP1-IL4-exo treatments control the numbers of monocytes and neutrophils in the circulation post-MI

**Figure 3:**
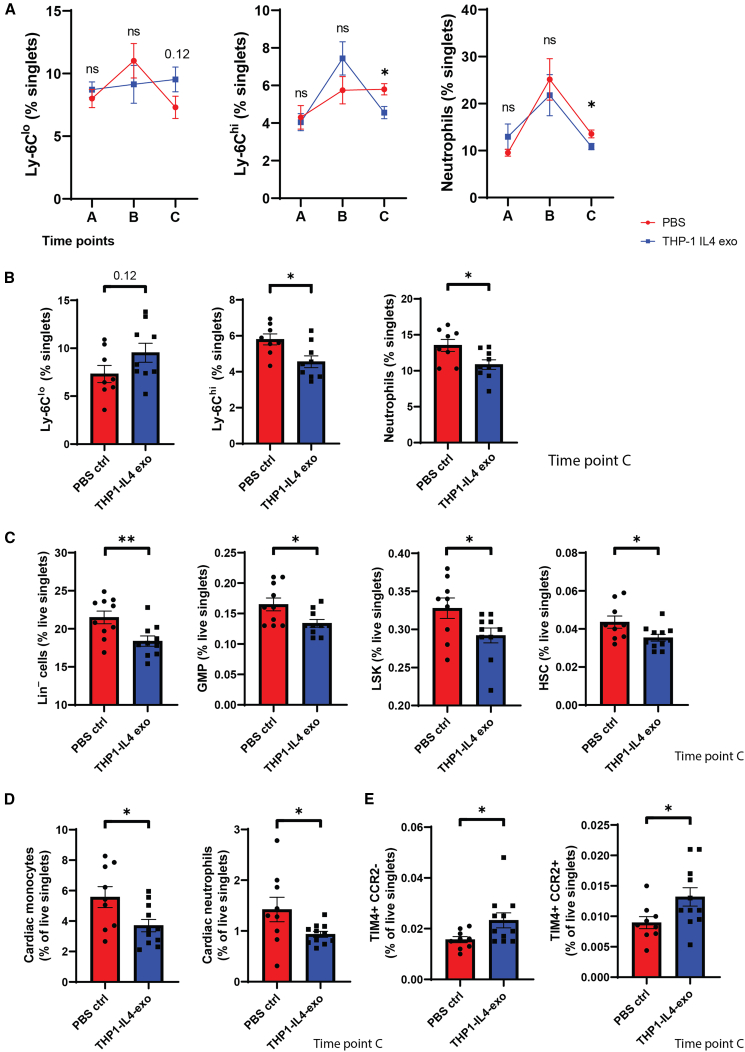
THP1-IL4-exo control the number of monocytes and neutrophils in the circulation, suppress hematopoiesis and myelopoiesis in the bone marrow, and suppress cardiac myeloid cell recruitment while preserving resident cardiac macrophage populations post-MI (A) Quantification of Ly-6C^lo^, Ly-6C^hi^, and neutrophil populations from the peripheral blood of mice treated with PBS vs. THP1-IL4-exo over the three time points A, B, and C; data pooled from two independent experiments; *n* = 8–9 per group. (B) Quantification of Ly-6C^lo^, Ly-6C^hi^, and neutrophil populations from the peripheral blood of mice treated with PBS vs. THP1-IL4-exo at time point C; data pooled from two independent experiments; *n* = 8–9 per group. (C) Quantification of lineage-negative (Lin^–^), granulocyte-monocyte progenitor (GMP), Lin^–^/Sca1^+^/c-Kit^+^ (LSK), and hematopoietic stem cell (HSC) populations from the bone marrow (BM) of mice treated with PBS vs. THP1-IL4-exo at time point C; data pooled from two independent experiments; *n* = 9–11 per group. (D) Quantification of cardiac monocytes and neutrophils from the digested hearts of mice treated with PBS vs. THP1-IL4-exo at time point C; data pooled from two independent experiments; *n* = 9–11 per group. (E) Quantification of TIM-4^+^/CCR2^–^ and TIM-4^+^/CCR2^+^ macrophage populations from the digested hearts of mice treated with PBS vs. THP1-IL4-exo at time point C; data pooled from two independent experiments; *n* = 9–11 per group. ∗*p* < 0.05 and ∗∗*p* < 0.01 as determined using unpaired Student’s *t* test. Data are represented as mean ± SEM.

Next, we examined the number of myeloid cells in the circulation, recognized for their propensity to traffic into the heart following MI.^7^^,^^8^^,^^13^^,^^29^ Figure S3A illustrates the gating strategy used to detect the numbers of circulating Ly-6C^lo^ and Ly-6C^hi^ monocytes, as well as neutrophils. No significant differences were observed among the three groups of myeloid cells in the circulation between time points A and B (Figure 3A). However, significant reductions in the numbers of circulating Ly-6C^hi^ and neutrophils were apparent in THP1-IL4-exo-treated mice at time point C, which also tended to display higher numbers of circulating Ly-6C^lo^ cells (Figure 3B). These data further support the benefits of systemic immunosuppression offered by the THP1-IL4-exo in this mouse model of hyperlipidemia-driven cardiovascular inflammation, where MI is recognized to contribute to myelopoiesis.^7^^,^^8^^,^^13^^,^^29^

### THP1-IL4-exo treatments suppress hematopoiesis and myelopoiesis in the BM post-MI

To further explore benefits of THP1-IL4-exo treatment in suppressing the number of inflammatory myeloid cells in the circulation, we examined populations of leukocyte progenitor cells in the BM. Figure S3B shows the gating strategy used to detect lineage-negative (Lin^–^) cells, granulocyte-monocyte progenitor (GMP) cells, Lin^–^/Sca-1^+^/c-Kit^+^ (LSK) cells, and hematopoietic stem cells (HSCs). Mice treated with THP1-IL4-exo displayed reduced populations of Lin^–^ cells, GMP cells, LSK cells, and HSCs (Figure 3C). Reduced hematopoiesis and myelopoiesis highlight the immunosuppressive properties of THP1-IL4-exo within the BM of mice that experienced MI, which mirrors our prior findings observed among obese diabetic mice with hyperlipidemia.^17^^,^^19^

### THP1-IL4-exo treatments suppress cardiac myeloid cell recruitment while preserving populations of resident cardiac macrophages post-MI

To investigate mechanisms through which treatments with THP1-IL4-exo controlled HFrEF, we tested their impact on modulating cardiac inflammation post-MI. Using the gating strategy shown in Figure S3C, we detected reduced numbers of monocytes and neutrophils in the cardiac tissue of THP1-IL4-exo treated mice (Figure 3D). Interestingly, THP1-IL4-exo treatments led to an increase in the number of resident cardiac macrophages (rcMacs), identified by the expression of the cell surface marker T cell membrane protein 4 (TIM-4^+^/CCR2^–^) (Figure 3E).^30^ Furthermore, recruited M2-like monocyte-derived macrophages positive for TIM-4 and CCR2 expression (TIM-4^+^/CCR2^+^) were also found to be more abundant in the cardiac tissue of mice treated with THP1-IL4-exo (Figure 3E). Together, these data demonstrate the efficacy of THP1-IL4-exo treatments in suppressing inflammation while augmenting the number of anti-inflammatory tissue-repair resident and monocyte-derived macrophages in the cardiac tissue of mice that develop HFrEF post-MI.

### THP1-IL4-exo treatments suppress type 1 IFN signaling along with other immunometabolic and inflammatory pathways in circulating Ly-6C^hi^ monocytes post-MI

**Figure 4:**
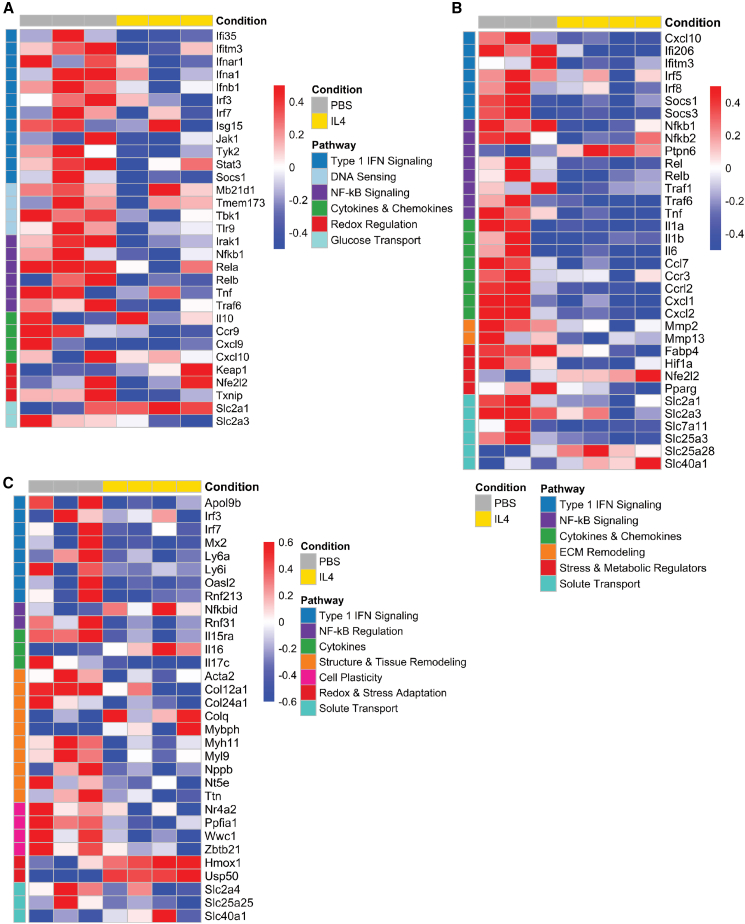
THP1-IL4-exo treatments suppress the type 1 interferon pathway in circulating Ly-6C^hi^ monocytes and CD11b^+^ myeloid cells from the BM and heart post-MI (A) NanoString transcriptomic analysis of Ly-6C^hi^ monocytes sorted from the peripheral blood of ApoE^h/h^/SR-B1^−/−^/Mx1-Cre^+^ mice treated with PBS vs. THP1-IL4-exo. The annotated heatmap illustrates the differential expression of genes associated with the type 1 interferon (IFN) pathway along with other inflammatory and metabolic pathways (*n* = 3). (B) Annotated differentially expressed gene (DEG) heatmap from unbiased RNA sequencing showing distinct mRNA expression profiles between the BM-derived CD11b^+^ myeloid cells of ApoE^h/h^/SR-B1^−/−^/Mx1-Cre^+^ mice treated with PBS vs. THP1-IL4-exo (*n* = 3–4 per group, raw *p* < 0.05). (C) Annotated DEG heatmap from unbiased RNA sequencing showing distinct mRNA expression profiles between cardiac-derived CD11b^+^ myeloid cells of ApoE^h/h^/SR-B1^−/−^/Mx1-Cre^+^ mice treated with PBS vs. THP1-IL4-exo (*n* = 3–4 per group, raw *p* < 0.05).

Because Ly-6C^hi^ monocytes are widely recognized to contribute to HF by driving inflammation, fibrosis, and adverse LV remodeling following MI,^31^ we sought to investigate their transcriptional responses following 4.5 weeks of THP1-IL4-exo treatments. The heatmap shown in Figure 4A highlights evidence of profound transcriptional reprogramming in Ly-6C^hi^ monocytes collected from both groups of mice. Notably, repeated THP1-IL4-exo treatments led to a downregulation of inflammatory genes within the type 1 IFN pathway, NF-κB-mediated signaling, and other inflammatory, metabolic, and stress-related pathways. Further support for a profound suppression of type 1 IFN inflammatory signaling is highlighted through reduced expression levels of the negative feedback regulator of inflammation, *Socs1*.^32^

### THP1-IL4-exo treatments control inflammation in the BM and heart post-MI via transcriptomic reprogramming of CD11b^+^ myeloid cells

**Figure 5:**
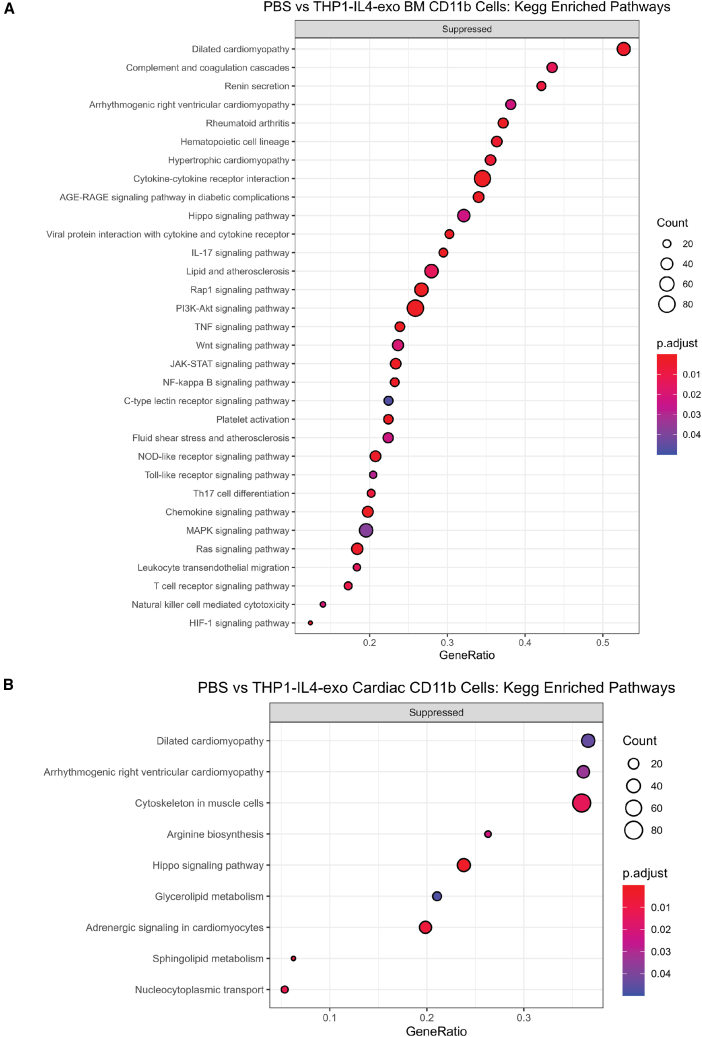
Inflammation control via THP1-IL4-exo revealed by RNA sequencing of CD11b^+^ myeloid cells collected from the BM and heart post-MI (A) Dot plot showing KEGG enrichment analysis using gene set enrichment analysis (GSEA) for DEGs from BM-derived CD11b^+^ myeloid cells of mice treated with PBS vs. THP1-IL4-exo. Suppressed pathways are shown. (B) Dot plot showing KEGG enrichment analysis using GSEA for DEGs from cardiac-derived CD11b^+^ myeloid cells of mice treated with PBS vs. THP1-IL4-exo. Suppressed pathways are shown.

To investigate the influence that 4.5 weeks of THP1-IL4-exo treatments imparted on transcriptional responses in myeloid cells residing in various tissues of ApoE^h/h^/SR-B1^−/−^/Mx1-Cre^+^ mice, CD11b^+^ cells were isolated from the BM and heart post-MI at time point C and subjected to unbiased RNA sequencing (RNA-seq) analysis. A heatmap of genes differentially expressed in CD11b^+^ cells isolated from the BM is shown in Figure 4B. Among these are genes encoding chemokines, inflammatory cytokines, and numerous type 1 IFN-related genes, including *Socs1*, findings that mirror the transcriptomic changes observed in Ly-6C^hi^ monocytes (Figure 4A). The transcriptome of CD11b^+^ cells isolated from cardiac tissue also support a robust suppression of inflammation with numerous ISGs, including *Irf7*, *Mx2*, and *Oasl2* all significantly suppressed in mice treated with THP1-IL4-exo (Figure 4C). Furthermore, KEGG Pathway analyses of transcriptional changes detected in CD11b^+^ myeloid cells derived from the BM and heart highlight that the control of inflammation by THP1-IL4-exo extends to the regulation of numerous pathways involved in atherosclerosis, such as lipid metabolism, fluid shear stress, and cytokine/cytokine-receptor interactions, including IFNs.^33^ Other major pathways modulated by THP1-IL4-exo include the Hippo signaling pathway, which is involved in cell death and survival, as well as pathways involved in cardiomyopathies (Figures 5A and 5B).^34^^,^^35^ Top immune pathways associated with type 1 IFN signaling, such as JAK-STAT,^36^ NF-κB,^37^ PI3K-Akt,^38^ and rheumatoid arthritis,^39^ were also significantly downregulated by THP1-IL4-exo. Together, the panoply of inflammatory genes and tissue remodeling pathways controlled by THP1-IL4-exo supports their capacity to broadly suppress inflammatory activities and adverse cardiac remodeling, contributing to the reversal of HF post-MI.

### THP1-IL4-exo treatments suppress the expression of genes responsive to type 1 IFN receptor signaling in the BM and heart post-MI

**Figure 6:**
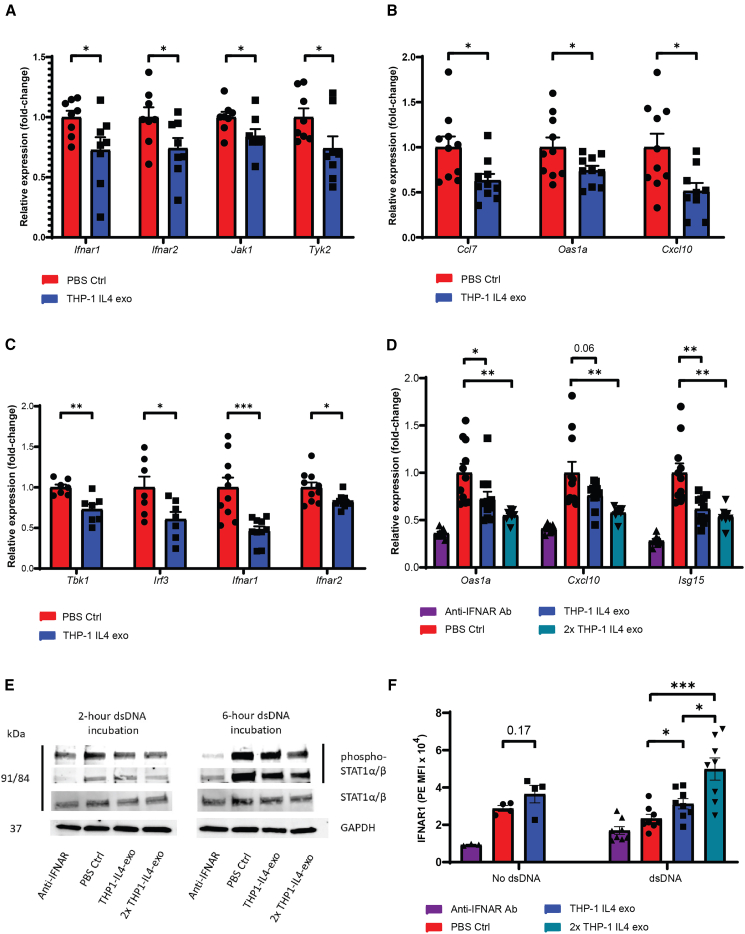
THP1-IL4-exo treatments suppress type 1 IFN signaling in CD11b^+^ myeloid cells of the BM and heart post-MI and in primary macrophages stimulated with dsDNA (A) RT-qPCR analysis of *Ifnar1*, *Ifnar2*, *Jak1*, and *Tyk2* mRNA expression in BM-derived CD11b^+^ myeloid cells from mice treated with PBS vs. THP1-IL4-exo. Gene expression was normalized to *B2m* and *Gapdh* mRNA and presented relative to control; data pooled from two independent experiments; *n* = 8 per group. (B) RT-qPCR analysis of *Ccl7*, *Oas1a*, and *Cxcl10* mRNA expression in whole-heart tissue from mice treated with PBS vs. THP1-IL4-exo. Gene expression was normalized to *B2m* and *Gapdh* mRNA and presented relative to control; data pooled from two independent experiments (*n* = 9–10 per group). (C) RT-qPCR analysis of *Tbk1*, *Irf3*, *Ifnar1*, and *Ifnar2* mRNA expression in cultured primary WT BMDM incubated with either PBS or THP1-IL4-exo, then transfected with dsDNA for 6 h. Gene expression was normalized to *B2m* and *Gapdh* mRNA and presented relative to control; data pooled from two independent experiments (*n* = 7 per group) or pooled from three independent experiments (*n* = 10 per group). (D) RT-qPCR analysis of *Oas1a*, *Cxcl10*, and *Isg15* mRNA expression in cultured primary WT BMDM incubated with anti-IFNAR neutralizing antibody, PBS, THP1-IL4-exo, or a 2× dose of THP1-IL4-exo, then transfected with dsDNA for 6 h. Gene expression was normalized to *B2m* and *Gapdh* mRNA and presented relative to control; data pooled from two independent experiments (*n* = 8 per group) or pooled from three independent experiments (*n* = 11 per group). (E) Western blot analysis of STAT1α/β and phosphorylated STAT1α/β protein levels in cell lysates of primary WT BMDM incubated with anti-IFNAR neutralizing antibody, PBS, THP1-IL4-exo, or a 2× dose of THP1-IL4-exo, then transfected with dsDNA for 2 or 6 h. (F) Graph showing mean fluorescence intensity of IFNAR1 signals in primary WT BMDM incubated with anti-IFNAR neutralizing antibody, PBS, THP1-IL4-exo, or a 2× dose of THP1-IL4-exo, then either transfected with dsDNA for 6 h or not; data for no dsDNA condition derived from one experiment (*n* = 3–4 per group); data for dsDNA condition pooled from two independent experiments (*n* = 8 per group). ∗*p* < 0.05, ∗∗*p* < 0.01, and ∗∗∗*p* < 0.001 as determined using unpaired Student’s *t* test. Data are represented as mean ± SEM.

Next, we sought to investigate mechanisms through which THP1-IL4-exo control type 1 IFN signaling in myeloid cells residing within the BM, recognized as a central source of inflammatory activity contributing to HF post-MI.^12^ Data shown in Figure 6A demonstrate a significant downregulation of the genes encoding the two subunits of IFNAR, a key mediator of type 1 IFN signaling, in CD11b^+^ cells isolated from the BM.^9^ Additionally, *Jak1* and *Tyk2* (Figure 6A), two genes encoding kinases responsible for signal transduction following IFNAR activation,^40^ were significantly downregulated. Data shown in Figure 6B highlight the capacity for THP1-IL4-exo to suppress the expression of ISGs in the heart, including *Ccl7*, *Oas1a*, and *Cxcl10*. Together, these data demonstrate the efficacy of THP1-IL4-exo treatments in suppressing type 1 IFN signaling in the BM and heart of mice post-MI.

### THP1-IL4-exo treatments suppress type 1 IFN receptor-mediated signaling in primary macrophages

The capacity for THP1-IL4-exo to control type 1 IFN-mediated inflammation was assessed in cultured primary wild-type (WT) mouse BM-derived macrophages (BMDMs) exposed to a 6-h pulse of dsDNA, a stimulus for cGAS-STING-mediated type 1 IFN signaling.^9^ Data shown in Figure 6C demonstrate that WT BMDM incubated with THP1-IL4-exo display the suppressed expression of genes upstream of the type 1 IFN-signaling pathway, *Tbk1* and *Irf3*, as well as the expression of *Ifnar1*/*2*. An exposure to THP1-IL4-exo also led to reduced expression of downstream ISGs following dsDNA incubation, which included reduced mRNA levels encoding *Oas1a*, *Cxcl10*, and *Isg15* (Figure 6D). Importantly, the data in Figure 6D reveal a dose-dependent effect of THP1-IL4-exo in mediating immunosuppressive properties, including a reduction in the expression of ISGs, similar to the effect observed with an IFNAR-neutralizing antibody.

Next, to explore the underlying mode of action that could account for the immunosuppressive properties of THP1-IL4-exo, we tested their impact in controlling JAK/STAT signaling, a necessary component recognized for propagating type 1 IFN inflammatory signaling and the downstream expression of ISGs.^40^^,^^41^ We did so by assessing levels of phosphorylated STAT1α/β protein in BMDM stimulated with dsDNA. Data shown in Figure 6E reveal the capacity for THP1-IL4-exo to decrease levels of phospho-STAT1α/β in a dose-dependent manner, mimicking responses seen with an IFNAR-neutralizing antibody.

Finally, we assessed the capacity of THP1-IL4-exo to modulate the dynamics of cell surface presence of the type 1 IFN receptor in primary BMDMs exposed to dsDNA. Data shown in Figure 6F reveal that WT BMDM treated with THP1-IL4-exo display increased IFNAR1 cell surface densities. Consistent with observations shown in Figures 6D and 6E, THP1-IL4-exo modulation of IFNAR1 cell surface densities was found to be dose dependent.

Our data introduce the therapeutic potential of THP1-IL4-exo, an M2-macrophage exosome, for the treatment of cardiac inflammation, adverse remodeling, and functional decline that develops subsequent to MI.

## Discussion

Despite decades of research pointing to the involvement of inflammation as a contributor to the pathogenesis of HFrEF post-MI,^7^^,^^29^ the use of anti-inflammatory drugs targeting NF-κB signaling have so far failed to show benefits in clinical outcomes.^42^ Thus, it remains uncertain whether immunomodulatory therapies could serve to effectively control HFrEF, which remains a significant source of premature morbidity and death.^43^ A promising approach could include the selective targeting of the innate immune pathway known as the cGAS-STING-IRF3-type 1 IFN axis, a DAMP sensing system that recognizes dsDNA released during ischemic cell death, a phenomenon common to numerous forms of CVD.^11^^,^^44^ Through a comprehensive series of studies, King et al. reported that interrupting type 1 IFN signaling via antibody-mediated targeting of IFNAR provides a therapeutic avenue to alleviate the onset and severity of HFrEF subsequent to MI.^9^

Building on our recent findings that introduced therapeutic benefits of M2-polarized macrophage exosomes in controlling cardiometabolic inflammation, insulin resistance, and atherosclerosis in obese diabetic mice,^17^^,^^19^ we tested their utility to control adverse cardiac remodeling and HF post-MI. With human translation in mind, we opted to study exosomes produced by the human monocytic cell line THP-1, as we recently reported.^17^

Our studies were performed using the ApoE^h/h^/SR-B1^−/−^/Mx1-Cre^+^ mouse model of diet-induced MI and HF that we developed and reported in prior studies.^20^^,^^21^ The model has since been used by other laboratories and is recognized as one that faithfully mimics human coronary heart disease and MI.^45^ In this model, expression of hypomorphic *Apoe*^h/h^ alleles, when paired with a loss of SR-B1 expression, provides a convenient platform to reproducibly generate mice with occlusive coronary atherosclerosis and ischemic myocardial disease within 4 weeks of initiating a diet rich in fat and cholesterol.^20^^,^^21^ Subsequent normalization of plasma cholesterol levels, achieved by returning the mice to a chow diet and inducing Cre-mediated repair of the HypoE alleles, enables a deceleration in atherosclerosis while prolonging survival, resulting in mice with ischemic myocardial injury. Mice that survive MI develop cardiac inflammation and soon thereafter, impaired LV function that models human HFrEF.^20^^,^^21^ Our prior studies of the model demonstrated the therapeutic utility of profound immunosuppression using the sphingosine 1 phosphate (S1P) analog FTY720 to improve survival and cardiac function among ApoE^h/h^/SR-B1^−/−^/Mx1-Cre^+^ mice with established ischemic cardiac disease.^20^^,^^21^ While outcomes of that study did not identify a molecular pathway responsible for the cardioprotective effects of FTY720, we now speculate that it could have originated from an attenuation of type 1 IFN signaling resulting from S1PR1-mediated suppression of IFNAR signaling,^46^ accounting for our reported observations of reduced levels of *Ccl7* and *Oas1a* mRNA expression in cardiac tissue.^21^

In this study, we uncovered that THP1-IL4-exo, a type of exosome produced by M2-polarized human macrophages, can serve as an effective cardioprotective agent in preventing cardiac functional decline and HF post-MI. Through serial echocardiographic measurements of cardiac performance in ApoE^h/h^/SR-B1^−/−^/Mx1-Cre^+^ mice that experienced diet-induced coronary atherosclerosis and cardiac ischemia, we recorded marked improvements in numerous parameters of cardiac LV function that included FS, EF, SV, and LVESV, all of which returned to pre-diet baseline levels as soon as 4 weeks of triweekly IP administrations of THP1-IL4-exo post-MI. Together, our findings demonstrate the potency of THP1-IL4-exo in protecting against the onset of HFrEF post-MI, while sham-treated mice experienced profound cardiac LV function decline.

Clues to appreciate the cardioprotective properties afforded by THP1-IL4-exo stem from our recent observations that reported their ability to suppress hematopoiesis and the number of monocytes and neutrophils in the circulation of mice with hyperlipidemia and diabetes.^17^^,^^19^ As noted in our prior studies, the control of myelopoiesis originated from within the BM, with robust decreases in the numbers of HSCs and a suppression in the granulocyte-monocyte progenitor lineage that serves as a source of myelopoiesis in response to hyperlipidemia, diabetes, and MI.^7^^,^^29^ THP1-IL4-exo were also effective in reducing the numbers of circulating Ly-6C^hi^ monocytes and neutrophils, recognized for their role in driving cardiac inflammation, while simultaneously raising levels of Ly-6C^lo^ monocytes that are increasingly recognized for their tissue-repair properties post-MI.^47^ The control of myelopoiesis subsequently led to reduced numbers of inflammatory cells recruited to the heart, supporting the benefits of THP1-IL4-exo in provoking general control of inflammation and myelopoiesis in mice that experience MI.

Beyond limiting the recruitment of inflammatory myeloid cells into the ischemic heart, THP1-IL4-exo promoted the differentiation of monocyte-derived macrophages into rcMacs, recognized as cells that express the TIM-4 cell-surface antigen.^30^^,^^48^ Indeed, TIM-4^+^ cardiac macrophages are recognized to exert profound anti-inflammatory properties that resolve cardiac inflammation, including through efferocytosis to clear apoptotic cells and cellular debris.^30^^,^^49^ Their augmented numbers in cardiac tissue of mice treated with THP1-IL4-exo likely contributed to our observed suppression of cardiac injury and the propagation of anti-inflammatory signals. Furthermore, THP1-IL4-exo promoted the retention of TIM-4^+^/CCR2^–^ rcMacs, a unique population of self-renewing myeloid cells recognized for protective functions that include the suppression of cardiac fibrosis, debris removal, and vascular and lymphatic remodeling in response to MI, even when present in small numbers.^30^^,^^50^ Together, our findings highlight the capacity for THP1-IL4-exo to effectively limit detrimental myeloid cell activity while promoting anti-inflammatory properties following ischemic myocardial injury caused by occlusive coronary atherosclerosis.

Insights to appreciate the therapeutic benefits exerted by THP1-IL4-exo emerged from a transcriptomic profiling of myeloid cells. Our findings identified type 1 IFN signaling as a dominant immune pathway controlled in Ly-6C^hi^ monocytes in the circulation, as well as in myeloid cells of the BM and cardiac tissue. Interestingly, the suppression of ISG transcripts in BM CD11b^+^ cells was less pronounced compared to circulating Ly-6C^hi^ monocytes or cardiac CD11b^+^ cells. Of these three tissues, the heart has been described as having the highest levels of ISG expression post-MI,^12^ and it also appeared to exhibit the greatest degree of responsiveness to THP1-IL4-exo-mediated control of ISG expression. However, beyond emulating results obtained using antibody-mediated blockade of INFAR,^9^ findings revealed through Kyoto Encyclopedia of Genes and Genomes (KEGG) Pathway analysis of our transcriptional data suggest an involvement of a constellation of other cell signaling and tissue repair properties inherent to THP1-IL4-exo that likely contributed to restore cardiac function in mice that experienced MI. Pathways that could have contributed to the improvement of cardiac healing could include those that we recently reported result in enhanced mitochondrial metabolism and oxidative phosphorylation, which are recognized to drive M2-like macrophage biology.^17^^,^^19^ Modulation of the genes responsible for regulating redox stress control, vascular repair, and suppression of genes contributing to adverse ventricular modeling, atherosclerosis, shear stress, and cardiomyopathies could have served to alleviate cardiac injury post-MI. These findings hint at the capacity of THP1-IL4-exo to impact a variety of distinct pathways that together drive the restoration of cardiac performance following ischemic myocardial injury.

While a complete understanding of the therapeutic benefits exerted by THP1-IL4-exo will require further study, it is likely that they derive in part from their ability to communicate microRNA to cardiac tissue, similar to what has been reported for miR-146a enriched in cardiosphere-cell-derived exosomes^51^ and miR-21 enriched exosomes produced by cultured mesenchymal stem cells.^52^^,^^53^ Interestingly, our prior findings documented the presence of these and other anti-inflammatory and metabolic microRNAs in THP1-IL4-exo.^17^ Furthermore, our *in vitro* data point to mechanisms that could account for cardioprotective properties of THP1-IL4-exo that likely stem from their ability to robustly polarize macrophages into an M2-like phenotype. Such benefits likely suppressed dsDNA-stimulated cGAS-STING activation, IFNAR signaling, and subsequent ISG gene expression and promoted the retention and accumulation of TIM-4^+^ rcMacs in cardiac tissue. The microRNA cargo in THP1-IL4-exo that we previously identified as central to immunosuppression and augmented mitochondrial metabolism include miR-146b/378a.^17^^,^^19^ Findings from this study identify an enrichment of miR-23a/b in THP1-IL4-exo that could have contributed to blunt cGAS-STING signaling,^25^ limiting type 1 IFN activation to attenuate cardiac injury post-MI. While the precise mechanism of action targeted by THP1-IL4-exo remains unclear, our data point to a dose-dependent control of STAT1 phosphorylation as a source of attenuated inflammatory gene transcription. This finding is consistent with our observations of reduced *Socs1* and *Irf5* in myeloid cells (Figures 4A and 4B), which are both responsive to STAT1 signaling^54^ and consistent with our prior results that demonstrate the capacity for THP1-IL4-exo to suppress inflammatory M1 polarization in macrophages.^17^^,^^19^ Further evidence supporting an attenuation of type 1 IFN signaling by THP1-IL4-exo derive from our observations of increased IFNAR1 cell surface density in primary macrophages stimulated with dsDNA. Indeed, productive IFNAR signaling is recognized to result in receptor internalization, ubiquitination, and subsequent degradation.^41^ Additional studies will be required to provide a more complete understanding of the inflammatory and metabolic networks that are subject to the control of THP1-IL4-exo. Such studies are warranted based on an increased interest in the study of the THP-1 cell line as a source of therapeutic exosomes for an increasing number of pathological disorders.^55^

Future studies will be required to investigate whether treatments with THP1-IL4-exo also benefit other cell types within the cardiovascular system, including cardiomyocytes, endothelial cells, and fibroblasts, to improve cardiac function. Furthermore, based on our observations demonstrating a capacity for THP1-IL4-exo to improve mitochondrial metabolism while controlling oxidative stress,^17^ similar benefits could have led to improved cardiomyocyte mitochondrial health and viability. Another interesting avenue of investigation would include addressing the benefits of reduced MMP expression by THP1-IL4-exo, notably *NTT-Mmp2*, which we previously identified as a source of innate immune activation contributing to adverse cardiac remodeling during ischemia.^26^ Additional studies will also be required to investigate the ability for THP1-IL4-exo to communicate benefits to atheroma in coronary arteries to drive the remodeling and stabilization of atherosclerotic plaques. Finally, it will be both interesting and of value to test whether THP1-IL4-exo can provide therapeutic benefits for other forms of HF, including those that develop subsequent to pulmonary hypertension, diabetic HF with preserved ejection fraction, and aging.

## Materials and methods

### Animal studies

ApoE^h/h^/SR-B1^−/−^ mice, which express an ApoE4-like form of murine ApoE known as R61 ApoE,^22^ were bred to Mx1-Cre transgenic mice, and the resulting offspring were used to conduct *in vivo* studies. At 20–24 weeks of age, ApoE^h/h^/SR-B1^−/−^/Mx1-Cre^+^ mice were fed Paigen’s Atherogenic Rodent Diet (Research Diets) for 4.5 weeks. This time point was chosen to induce occlusive coronary atherosclerosis, MI, and HF in the mice, as we previously reported.^20^^,^^21^ Subsequently, 250 μg pIpC dissolved in 100 μL saline was injected into each mouse over the course of 2 days, and mice were also switched to a chow diet (Teklad). Caloric restriction and pIpC-mediated repair of ApoE resulted in robust plasma lipid lowering. During this time, cohorts of mice were also injected three times per week with either 10^10^ particles of THP1-IL4-exo or PBS control. This treatment paradigm continued for 4.5 weeks. Data collection and analyses were conducted in a blinded fashion. All mice were housed and bred in specific pathogen-free conditions in the Animal Research Facility at the San Francisco Veterans Affairs Medical Center. All animal experiments were approved by the Institutional Animal Care and Use Committee at the San Francisco Veterans Affairs Medical Center.

### Measurement of cholesterol levels in mouse plasma

Paigen diet-fed ApoE^h/h^/SR-B1^−/−^/Mx1-Cre^+^ mice that were treated three times per week with PBS vs. THP1-IL4-exo were anesthetized with isoflurane (Baxter), after which their blood was collected via retro-orbital puncture with micro-hematocrit capillary tubes (Fisher Scientific) and placed into tubes with 0.5 M EDTA (Invitrogen). Peripheral blood was spun at 4,000 rpm for 30 min at 4°C to collect the plasma. Plasma cholesterol was measured using the Cholesterol E Assay Kit (FUJIFULM Wako Pure Chemical Corporation).

### THP-1 cell culture and production of IL-4 conditioned media

The human monocytic cell line THP-1 was purchased from the University of California, San Francisco (UCSF) Cell and Genome Engineering Core as an authenticated stock. Cells were cultured in RPMI 1640 media (Corning) supplemented with 10% fetal bovine serum (Gibco), 1% GlutaMAX (Gibco), and 1% penicillin-streptomycin (Gibco). THP-1 cells were grown in a T-25 flask and expanded in a T-75 flask (Thermo Fisher Scientific) until they reached a density of 10^6^ cells/mL. Once confluent, cells were seeded into 100-mm plates (VWR) at 4 × 10^6^ cells/plate. Phorbol 12-myristate 13-acetate (PMA; Thermo Fisher Scientific) was added to the plates at a concentration of 25 ng/mL for 48 h, differentiating the THP-1 cells into macrophages. THP-1 macrophages were then cultured in PMA-free media for an additional 72 h, followed by culturing in exosome-free media (EFM) supplemented with 20 ng/mL of recombinant human IL-4 (PeproTech) to produce IL-4 conditioned media (CM).^17^

### THP-1 macrophage exosome isolation and nanoparticle tracking analysis

Exosome isolation and characterization experiments were performed in accordance with the Minimal Information for Studies of Extracellular Vesicles (MISEV) 2023 guidelines.^56^ After growing the differentiated THP-1 macrophages in dishes with PMA-free media for 72 h, the media was removed, the plates were washed with PBS (Corning) twice, and EFM supplemented with IL-4 was added for 24 h. The CM was collected the following day and centrifuged at 400 × *g* for 10 min, poured into a new tube, centrifuged at 2,000 × *g* for 20 min, and filtered with a 0.2-μm membrane. THP1-IL4-exo were then isolated using C-DGUC.^23^^,^^24^ The filtered CM was first centrifuged on a 60% OptiPrep cushion (STEMCELL Technologies) at 100,000 × *g* for 3 h and at 4°C (Type 45 Ti, Beckman Coulter) to concentrate the exosomes. Afterward, the cushion was collected and centrifuged along with a 5%, 10%, and 20% OptiPrep density gradient at 100,000 × *g* overnight and at 4°C (SW 40 Ti, Beckman Coulter). The following day, twelve 1-mL fractions were collected, starting from the top and following the meniscus. Fraction 7 from the density gradient was dialyzed in PBS using the Slide-A-Lyzer MINI Dialysis Device (Thermo Fisher Scientific) and used for characterization, *in vitro*, and *in vivo* experiments.

The vesicles in fraction 7 were subjected to nanoparticle tracking analysis (NTA) using the NanoSight LM10 (Malvern Panalytical), which provided size and concentration data. Samples were diluted in 1:100 PBS and measured in triplicate. The analysis settings were optimized and kept identical for each sample, and data were analyzed using the NanoSight NTA version 3.3 software (Malvern Panalytical). All exosome samples were stored at 4°C and used after isolation or stored at −80°C.

### Transmission electron microscopy

Exosome morphology was assessed via transmission electron microscopy by loading 7 × 10^8^ exosomes onto a glow-discharged 400-mesh Formvar-coated copper grid (Electron Microscopy Sciences). The exosomes were left to settle for 2 min, after which the grids were washed four times with 1% uranyl acetate. Excess uranyl acetate was blotted off gently with filter paper. Grids were then dried and imaged at 120 kV using a Tecnai 12 transmission electron microscope (FEI).

### Labeling and *in vitro* tracking of THP1-IL4-exo biodistribution

THP1-IL4-exo were labeled with DiR (DilC18(7); Invitrogen). The dye was added to the 60% OptiPrep cushion at a concentration of 10 μM, incubated for 20 min, and then isolated following the same C-DGUC protocol as previously described. After dialysis and NTA characterization, 10^10^ particles of DiR-labeled THP1-IL4-exo or an equivalent volume of PBS was injected IP into ApoE^h/h^/SR-B1^−/−^/Mx1-Cre^+^ mice that had been fed a Paigen diet for 4.5 weeks, induced with pIpC, and returned to a chow diet. After 6 h, the mice were sacrificed, perfused with PBS, and their blood, tibias, femurs, and hearts were collected. The DiR fluorescence signal was imaged using the Odyssey CLx (LI-COR Biosciences) imaging system and analyzed using the Image Studio software (LI-COR Biosciences ).

### Echocardiographic analysis of heart function

Transthoracic echocardiography was performed with a Vevo F2 (FUJIFILM VisualSonics) system using the UHF46x 46-20 MHz transducer. Mice were lightly sedated with isoflurane (Baxter), shaved, secured to a heating platform in a supine position, and monitored for body temperature and consistent heart rate. Ultrasound gel (Aquasonic) was applied to the shaved chest. Two-dimensional (2D) long-axis images of the LV were recorded at the plane of the aortic and mitral valves, where the LV cavity is largest. Additionally, 2D short-axis images were obtained at the papillary muscle level. All measurements were derived from digital images capture on cine loops at time points A, B, and C. The Vevo Lab analysis software (FUJIFILM VisualSonics) was used to analyze data for cardiac function that included EF, FS, LVESV, and SV.

### RNA extraction and gene expression analysis using RT-qPCR

Cohorts of PBS control (ctrl) vs. THP1-IL4-exo-treated mice were sacrificed and perfused with PBS. Hearts were collected and homogenized in QIAzol with a Tissue-Tearor homogenizer (BioSpec). Total RNA from cells in QIAzol was then extracted using the RNeasy Mini Kit (QIAGEN) according to the manufacturer’s protocol. RNA was then quantified using NanoDrop, and 300 ng RNA served for the synthesis of cDNA using the iScript Reverse Transcription Supermix (Bio-Rad). qPCR reactions were performed using the iTaq Universal SYBR Green Supermix (Bio-Rad) and run on the CFX Opus 384 Real-Time PCR System (Bio-Rad). Cycle threshold values were normalized to the housekeeping genes *B2m* and *Gapdh* and analyzed using Bio-Rad CFX Maestro software. All reactions were performed in triplicate. RNA extracted from CD11b^+^ myeloid cells of both the BM and heart tissue was also subjected to gene expression analysis via RT-qPCR, following the protocol as previously described.

### Circulating and tissue-associated leukocyte detection using flow cytometry

Peripheral blood was collected from cohorts of PBS ctrl vs. THP1-IL4-exo-treated mice at time points A, B, and C as previously described. Red blood cells (RBCs) were lysed with 1× RBC Lysis Buffer (BioLegend). Nonspecific binding was blocked using TruStain FcX anti-mouse CD16/32 antibodies (BioLegend) for 10 min at 4°C in fluorescence-activated cell sorting (FACS) buffer, followed by staining with anti-CD45 (clone 30-F11) and anti-CD11b (clone M1/70), anti-CD115 (clone AFS98), and anti-Ly-6C (clone HK1.4; all clones from BioLegend) for 30 min at 4°C and in the dark. This allowed for the detection of circulating Ly-6C^lo^, Ly-6C^hi^, and neutrophils. The antibody dilutions ranged from 1:100 to 1:200.

For the BM, cohorts of PBS ctrl vs. THP1-IL4-exo-treated mice were sacrificed and perfused with PBS. Subsequently, their tibias, femurs, and hearts were collected. Tibias and femurs were flushed by centrifugation at 3,000 × *g* for 3 min to obtain the cells of the BM. RBCs were lysed with 1× RBC Lysis Buffer (BioLegend). Cells were then stained in FACS buffer containing a lineage-marker mix of biotinylated anti-CD4 (clone RM4.5), anti-CD8 (clone 53-6.7), anti-CD45R/B220 (clone RA3-6B2), anti-TER-119 (clone TER-119), anti-Gr-1 (clone RB6-8C5), and anti-CD127 (clone A7R34; all 6 clones from BioLegend) for 30 min at 4°C and in the dark. Afterward, cells were stained with anti-CD34 (clone RAM34), anti-CD48 (clone HM48.1), anti-Ly-6A/E/Sca-1 (clone D7), anti-CD135 (clone A2F10), anti-CD117/c-Kit (clone 2B8; all 5 clones from Invitrogen eBioscience), anti-CD150/SLAM (clone TC15-12F12.2), anti-CD16/32 (clone 93), anti-CD41 (clone MWReg30; all 3 clones from BioLegend), and streptavidin (clone 563858; BD Biosciences) for 90 min at 4°C and in the dark. Cells were gently mixed every 20 min, which allowed for the detection of hematopoietic stem and progenitor cell populations. The antibody dilutions ranged from 1:100 to 1:200.

For hearts, cohorts of PBS ctrl vs. THP1-IL4-exo-treated mice were sacrificed and perfused with PBS. Hearts were collected and digested using the Multi Tissue Dissociation Kit (Miltenyi Biotec) and the gentleMACS Octo Dissociator (Miltenyi Biotec), according to the manufacturer’s protocol. RBCs were then lysed with 1× RBC Lysis Buffer (BioLegend). Nonspecific binding was blocked using TruStain FcX anti-mouse CD16/32 antibodies (BioLegend) for 10 min at 4°C in FACS buffer, followed by staining with anti-CD45 (clone 30-F11), anti-CD11b (clone M1/70), anti-CD64 (clone X54-5/7.1), anti-Ly-6C (clone HK1.4), anti-Ly-6G (clone HK1.4), anti-CD119/CCR2 (clone SA20361.1), and anti-TIM-4 (clone RMT4-54; all clones from BioLegend) for 30 min at 4°C and in the dark. This allowed for the detection of cardiac monocytes, neutrophils, and rcMacs. The antibody dilutions ranged from 1:100 to 1:200.

All flow cytometric analyses were performed using a CytoFLEX S Flow Cytometer (Beckman Coulter), and data were collected and analyzed using FlowJo version 10.9.0.

### Transcriptional profiling of gene expression in circulating Ly-6C^hi^ monocytes

Blood was collected, processed, and stained as previously described. Ly-6C^hi^ monocytes were sorted using the BD FACSAria Fusion (BD Biosciences). RNA was isolated from the sorted Ly-6C^hi^ monocytes using the RNeasy Mini Kit (QIAGEN), quantified using the Quant-iT RiboGreen RNA Assay Kit, and 10 ng RNA was amplified using the nCounter Low RNA Input Amplification Kit before expression analysis with the nCounter analysis system (NanoString Technologies). The mRNA detection procedure was performed according to the manufacturer’s protocol using the autoimmune profiling panel for mice. The number of mRNA molecules counted was imported into the NSolver 4.0 (NanoString Technologies), with default settings, and corrected and normalized against the reference genes annotated in the kit that were found to be stable. The software generated an advanced analysis report, and results were considered statistically significant when *p* ≤ 0.05. Heatmaps were created using the pheatmap (version 1.0.10) package in R.

### Isolation of CD11b^+^ myeloid cells from mouse BM and heart for RNA-seq and RT-qPCR gene analysis

Cells were isolated from the tibias, femurs, and hearts of PBS ctrl vs. THP1-IL4-exo-treated mice as previously described. After achieving a cell suspension of BM or cardiac cells, both cell suspensions were subject to 1× RBC Lysis Buffer (BioLegend), followed by the isolation of CD11b^+^ cells using mouse CD11b magnetic MicroBeads (Miltenyi Biotec), performed according to the manufacturer’s protocol. Cells were then placed in QIAzol Lysis Reagent (QIAGEN). RNA was extracted from cells using the RNeasy Mini Kit (QIAGEN) according to the manufacturer’s protocol. Extracted RNA was subjected to RT-qPCR analysis following the protocol as previously described or to RNA-seq transcriptomic analysis.

Isolated RNA was measured for quantity with Quant-iT Ribogreen RNA Assay (Thermo Fisher Scientific) and quality with Agilent High Sensitivity RNA Screen Tape and buffer (Agilent Technologies). For mouse RNA samples, an indexed, Illumina-compatible, double-stranded cDNA whole-transcriptome library was synthesized from 10 ng total RNA with Takara Bio’s SMARTer Stranded Total RNA-Seq kit v2 Pico Input Mammalian and their SMARTer RNA Unique Dual Index Kit. Library preparation included RNA fragmentation (94°C for 4 min), cDNA synthesis, a 5-cycle indexing PCR, ribosomal cDNA depletion, and a 12-cycle enrichment PCR. Each library was measured for size with Agilent Technologies’ High Sensitivity D1000 ScreenTape and reagents and concentration with KAPA SYBR FAST Universal qPCR Kit (Kapa Biosystems). Libraries were then combined into an equimolar pool, which was also measured for size and concentration. The pool was clustered onto a flowcell (Illumina) with a 1% v/v PhiX Control v3 spike-in (Illumina) and sequenced on Illumina’s NovaSeq 6000 at a final flowcell concentration of 400 pM. The first and second reads were each 100 bases.

For data processing, the SMARTer Total RNA Pico v2 reads are quality filtered and trimmed as recommended by Takara Bio with the removal of the first 3 bases of read2. After trimming and filtering reads are genome and transcriptome mapped using STAR (version 2.5.3a). Aligned BAM files are converted into gene counts matrices for further analysis using FeatureCounts (version 2.0.1), using read2 as the sense strand. For RNA-seq analysis, differential expression was conducted using the DESeq2 package (version 1.20.0) in R (version 3.5.0) for all gene expression analyses. The raw read counts for the samples were normalized using the median ratio method (default in DESeq2). The significant differentially expressed genes (by Benjamini-Hochberg adjusted *p* values) are reported in the paper. Heatmaps were created using the pheatmap (version 1.0.10) package in R.

### Testing type 1 IFN signaling in cultured WT BMDM in response to dsDNA

WT murine BMDMs were derived and cultured as previously described.^17^^,^^18^^,^^19^ Briefly, BM cells were flushed from the tibia and femurs of 6- to 12-week-old WT mice on C57BL/6J background. Cells were cultured in complete media containing DMEM (Corning) supplemented with 10% fetal bovine serum (Gibco), 1% GlutaMax (Gibco), and 1% penicillin-streptomycin (Gibco) and differentiated with 25 ng/mL mouse macrophage-colony-stimulating factor (PeproTech) for 6 days in 37°C and 5% CO_2_. Confluent WT BMDMs were then seeded into 6-well culture plates (Corning) at a concentration of 1 × 10^6^ cells/well and incubated with 10^10^ particles/mL of THP1-IL4-exo or an equal volume of PBS for 18 h. The following day, cells designated for the anti-IFNAR antibody condition were incubated with 20 μg/mL of an IFNAR neutralizing antibody (clone MAR1-5A3, BioXCell) for 2 h. Cells designated for a 2× dose of THP1-IL4-exo were supplemented with an additional 10^10^ particles/mL of THP1-IL4-exo. Cells were then transfected with jetPRIME transfection reagent (Polyplus) and 2 μg/mL dsDNA for 2 or 6 h according to the manufacturer’s protocol. For this experiment, dsDNA was produced by annealing single-stranded DNA (Integrated DNA Technologies) as reported by Chamma et al.^57^

After dsDNA incubation, cells were subjected to RT-qPCR or western blot analyses. RNA collection and RT-qPCR analysis were conducted following the protocol as previously reported. For western blot analysis, cells were lysed with radioimmunoprecipitation assay buffer (Thermo Fisher Scientific) supplemented with 0.5 M EDTA (Thermo Fisher Scientific) and Halt Protease & Phosphatase Inhibitor Cocktail (Thermo Fisher Scientific) and then sonicated. Protein concentrations were measured using the Pierce BCA Protein Assay kit (Thermo Fisher Scientific). A total of 10 μg protein was diluted with PBS to 37.5 μL and then mixed with 12.5 μL 4× Laemmli buffer (Bio-Rad) and 10% 2-mercaptoethanol (Sigma-Aldrich). Samples were subsequently heated at 95°C for 5 min. Samples were then loaded on a 4%–20% Mini-PROTEAN TGX polyacrylamide gel (Bio-Rad) and transferred onto a polyvinylidene fluoride membrane (Bio-Rad). The membranes were blocked with 5% BSA (Sigma-Aldrich) in Tris-buffered saline (TBS) (Fisher Scientific) for 1 h and then incubated with primary antibodies overnight at 4°C. Primary antibodies for cell lysates of primary BMDM include anti-STAT1 (1:1,000, Cell Signaling Technology), anti-phospho-STAT1 (1:500, Cell Signaling Technology), and anti-glyceraldehyde 3-phosphate dehydrogenase (1:500, Santa Cruz Biotechnology). After four washes in TBS containing 0.01% Tween (TBS-T), membranes were incubated with corresponding horseradish peroxidase (HRP)-conjugated secondary antibodies: anti-rabbit immunoglobulin G (IgG) HRP (1:2,000, Invitrogen) or anti-mouse IgG_1_ HRP (1:1,000, Santa Cruz Biotechnology) for 1 h and washed in TBS-T. Signals were visualized after incubation with Clarity Western ECL Substrate (Bio-Rad) and imaged using an ImageQuant LAS 4000 (GE Healthcare).

### Quantification and statistical analysis

Statistical analyses were performed using GraphPad Prism 8, using the unpaired, two-tailed Student’s *t* test (two groups); ∗*p* < 0.05; ∗∗*p* < 0.01; ∗∗∗*p* < 0.001; ∗∗∗∗*p* < 0.0001. All error bars represent the mean ± standard error of the mean (SEM). All experiments were repeated at least twice or performed with independent samples.

## Data and code availability

All data presented in the main text or the supplemental information are available upon request.

## Acknowledgments

We thank the staff of the Electron Microscope Laboratory at the University of California, Berkeley for advice and assistance with electron microscopy sample preparation and imaging. We also thank the UCSF Genomics CoLab facility for assistance with RNA-seq experiments and data analysis. This study was supported by grants to R.L.R. from the 10.13039/100000738Department of Veterans Affairs Merit Grant (no. I01BX003928), the 10.13039/100000738Department of Veterans Affairs Merit Grant (no. I01BX003928 BRAVE supplement), the Department of Veterans Affairs Research Career Scientist Award (no. IK6BX005692), and the 10.13039/100000002National Institutes of Health (no. UH3CA241703). The graphical abstract and Figure S1 were created using Biorender.

## Author contributions

Conceptualization, M.N. and R.L.R.; methodology, M.N., A.S.G., and R.L.R.; investigation, M.N., A.S.G., T.A.P., N.K.V., and R.L.R.; visualization, M.N., A.S.G., and T.A.P.; funding acquisition, R.L.R.; project administration, R.L.R.; supervision, R.L.R.; writing – original draft, M.N. and R.L.R.; writing – review & editing, M.N., A.S.G., T.A.P., N.K.V., and R.L.R.

## Declaration of interests

M.N., T.A.P., N.K.V., and R.L.R. have filed an invention disclosure related to some aspects of this work with the University of California, San Francisco, and the US Department of Veterans Affairs.

## Supplemental information

Document S1. Figures S1–S3

Document S2. Article plus supplemental information
