# Supplementary material for: M2 macrophage exosomes reverse heart failure post-myocardial infarction by suppressing type 1 interferon signaling in myeloid cells
Source: Mol Ther. 2025 Oct 4;34(1):494–510. doi: 10.1016/j.ymthe.2025.10.010 (PMC12925770; doi:10.1016/j.ymthe.2025.10.010)
Supplement: Document S1. Figures S1–S3 [file mmc1.pdf]

YMTHE, Volume *34*

Supplemental Information

M2 macrophage exosomes reverse heart failure post-myocardial infarction by suppressing type 1 interferon signaling in myeloid cells

Martin Ng, Alex S. Gao, Tuan Anh Phu, Ngan K. Vu, and Robert L. Raffai

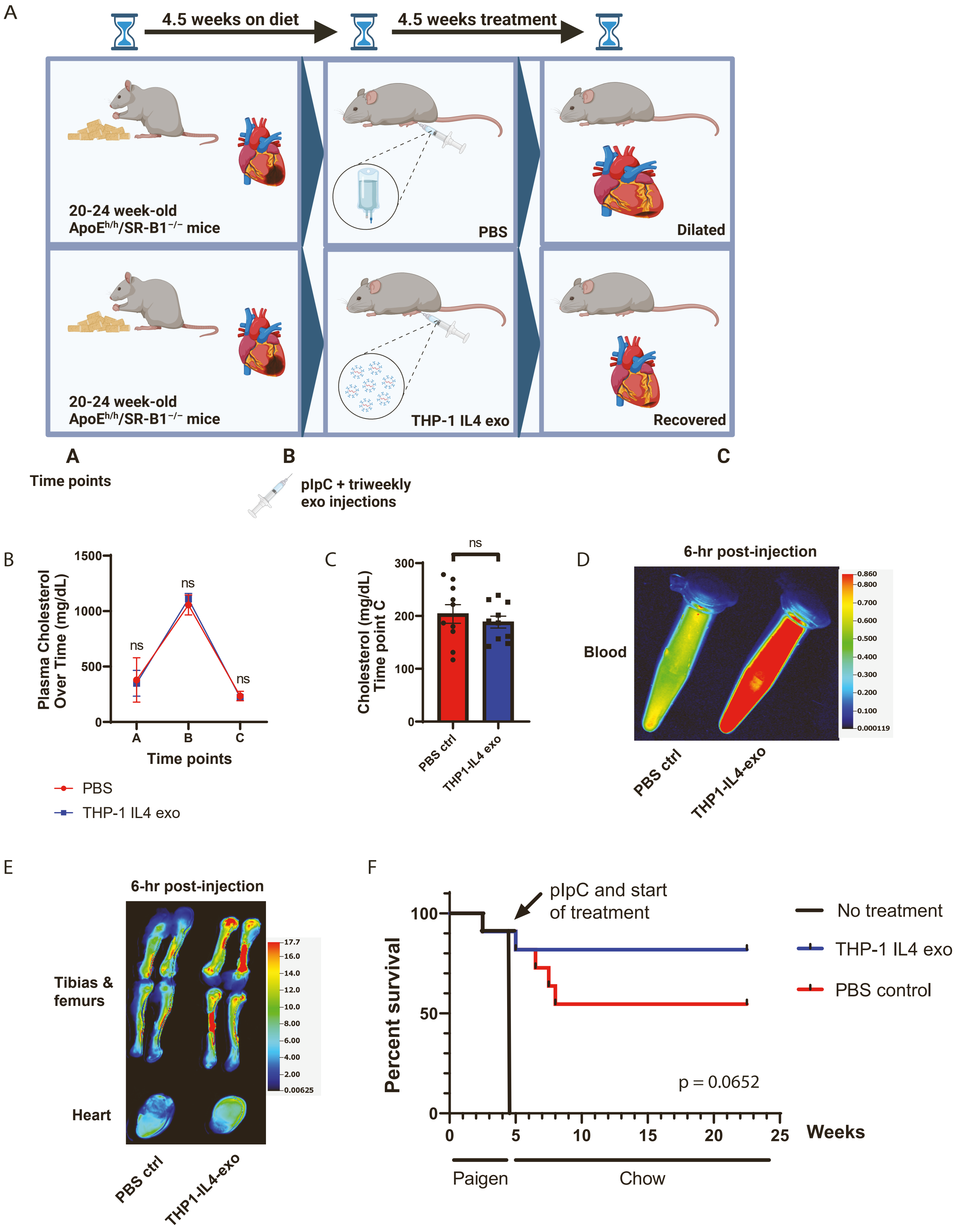
A
4.5 weeks on diet
4.5 weeks treatment
20-24 week-old
ApoE$^{h/h}$/SR-B1$^{-/-}$ mice
PBS
Dilated
20-24 week-old
ApoE$^{h/h}$/SR-B1$^{-/-}$ mice
THP-1 IL4 exo
Recovered
A
Time points
B
pIpC + triweekly
exo injections
C
B
Plasma Cholesterol
Over Time (mg/dL)
1500
1000
500
0
ns
ns
ns
A
B
C
Time points
PBS
THP-1 IL4 exo
C
Cholesterol (mg/dL)
Time point C
300
200
100
0
ns
PBS ctrl
THP1-IL4 exo
D
6-hr post-injection
Blood
0.860
0.800
0.700
0.600
0.500
0.400
0.300
0.200
0.100
0.000119
PBS ctrl
THP1-IL4-exo
E
6-hr post-injection
Tibias &
femurs
Heart
17.7
16.0
14.0
12.0
10.0
8.00
6.00
4.00
2.00
0.00625
PBS ctrl
THP1-IL4-exo
F
pIpC and start
of treatment
100
50
0
Percent survival
No treatment
THP-1 IL4 exo
PBS control
p = 0.0652
0
5
10
15
20
25
Weeks
Paigen
Chow

Figure S1. Study design utilizing ApoE$^{h/h}$/SR-B1$^{-/-}$Mx1-Cre^{+} mice

(A) Schematic depicting the time course involving diet modification, plasma lipid lowering, and exosome treatments. Time point A corresponds to the period before starting Paigen's atherogenic diet. Time point B occurs when mice have been on the diet for 4.5 weeks. Time point C occurs when mice have experienced tri-weekly treatments of PBS vs THP1-IL4-exo for 4.5 weeks.

(B) Plasma cholesterol levels in both treatment groups at Time point A, B, and C; n = 5 per group.

(C) Plasma cholesterol levels at Time point C; data pooled from two independent experiments; n = 10 per group.

(D and E) Images of DiR fluorescence in the blood (D), tibias, femurs, and hearts (E) 6 hr post-injection from ApoE$^{h/h}$/SR-B1$^{-/-}$Mx1-Cre^{+} mice that had been fed a Paigen's diet for 4.5 weeks, induced with pIpC, returned to a chow diet, and injected IP with PBS or 10^{10} particles of DiR-labeled THP1-IL4-exo.

(F) Kaplan-Meier survival curve of ApoE$^{h/h}$/SR-B1$^{-/-}$Mx1-Cre^{+} mice fed Paigen diet followed by a pIpC injection and chow diet, treated with PBS vs THP1-IL4-exo for up to 23 weeks. $p < 0.0652$ as determined by a log-rank test; n = 10 per group.

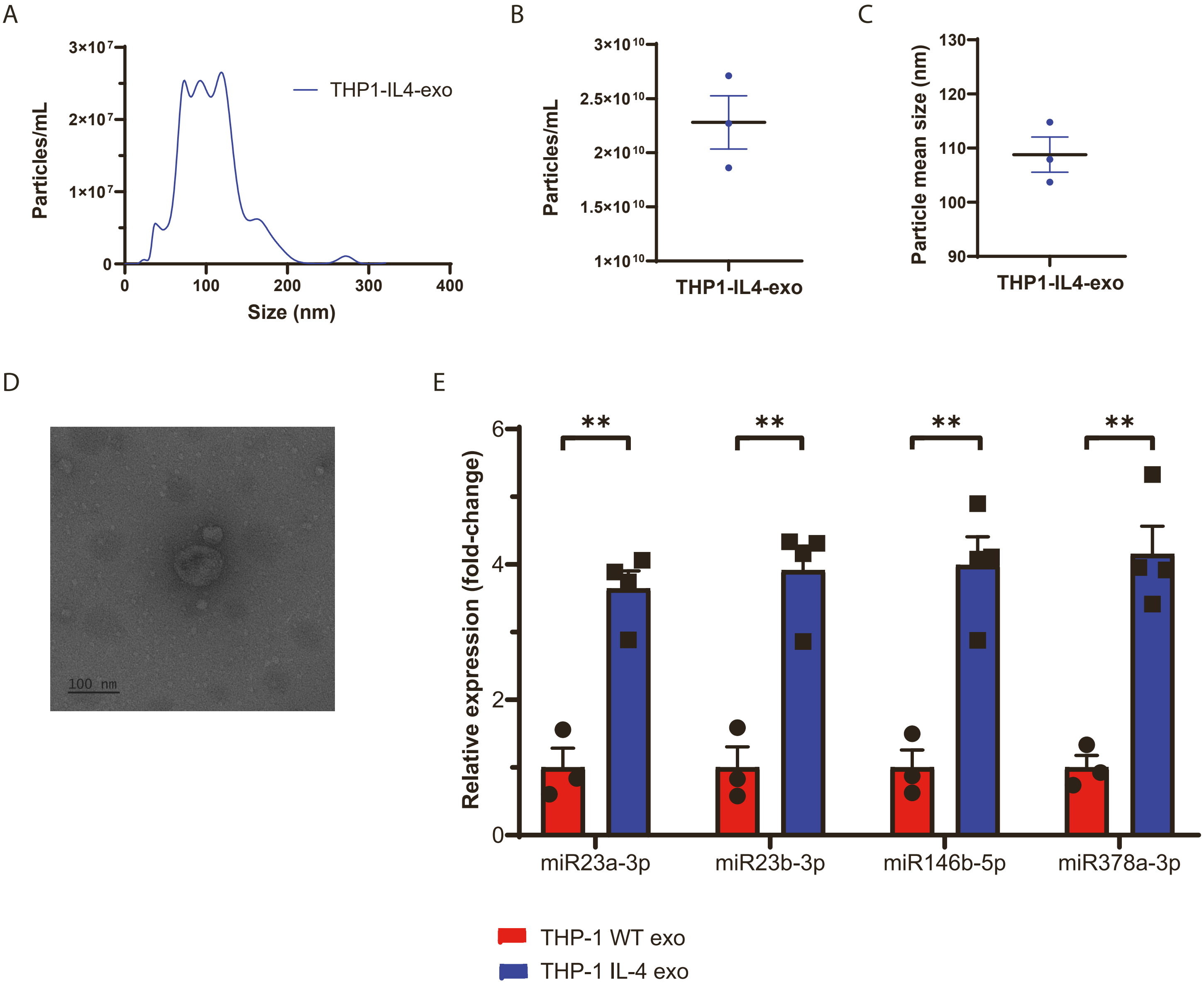

Figure S2. Biophysical parameters of THP1-IL4-exo

(A) Representative concentrations and size distributions of THP1-IL4-exo purified from THP-1 conditioned cell culture supernatant after a 24 hr culture period with IL4-supplemented exosome-free media determined using Nanoparticle Tracking Analysis (NTA).

(B and C) Average particle concentration per milliliter (B) and mean diameter in nanometers (C) determined using NTA; n = 3.

(D) Electron micrograph depicting a single purified THP1-IL4-exo in the center; scale bar: 100nm.

(E) RT-qPCR analysis of miR-23a-3p, -23b-3p, -146b-5p, and -378a-3p microRNA levels in THP-WT-exo vs THP1-IL4-exo. Results were normalized to U6 snRNA and UniSp6 (spike-in control). Separate preparations of exosomes were analyzed; n = 3-4 per group.

*p < 0.05 and **p < 0.01 as determined using unpaired Student's t test. Data are represented as mean ± SEM.

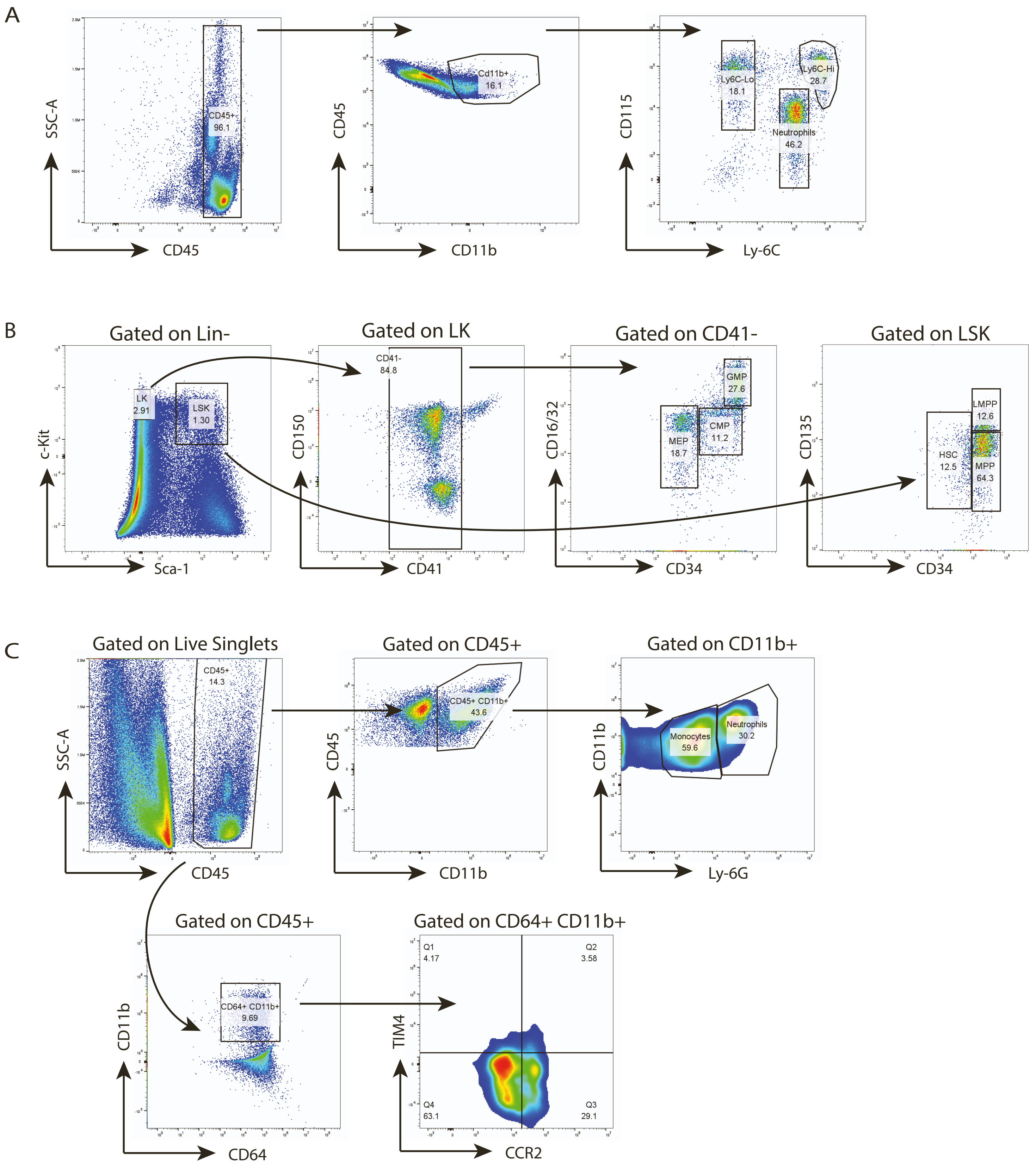

Figure S3. Flow cytometry gating strategies

(A) Representative flow cytometry plots of leukocyte subsets from peripheral blood.

(B) Representative flow cytometry plots of leukocyte progenitor subsets from bone marrow.

(C) Representative flow cytometry plots of leukocyte subsets from digested whole heart tissues.
